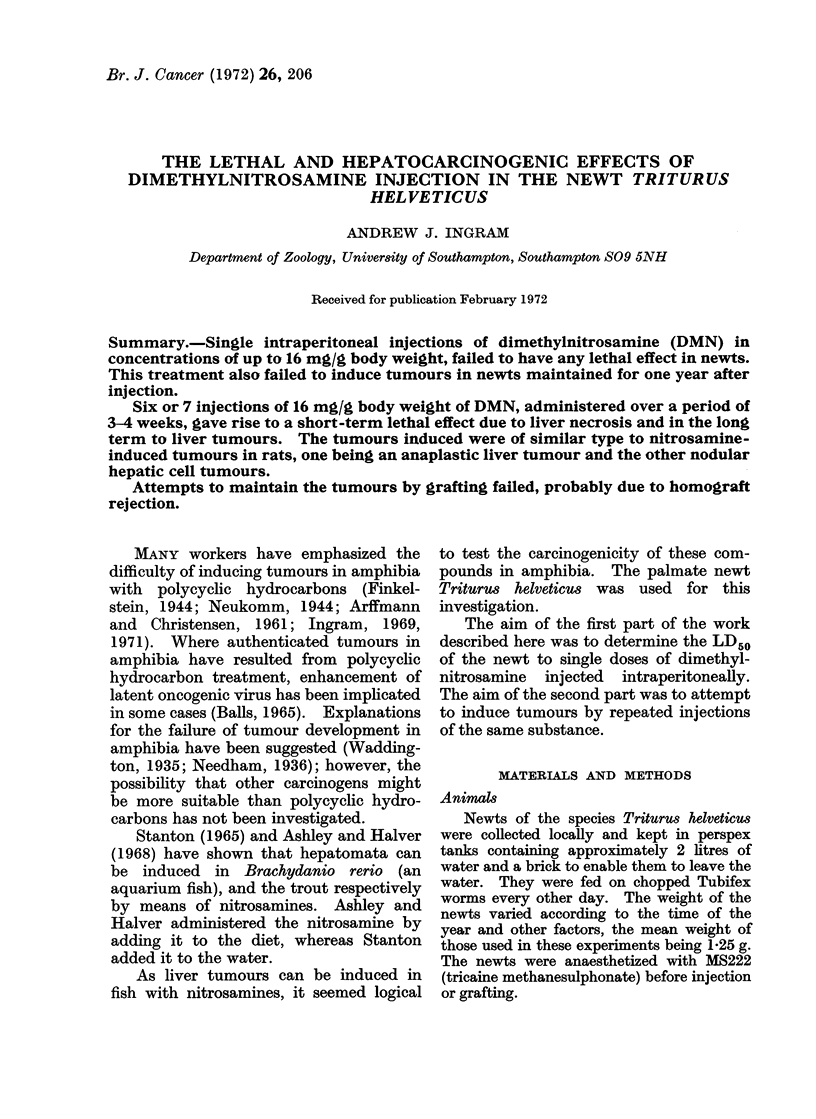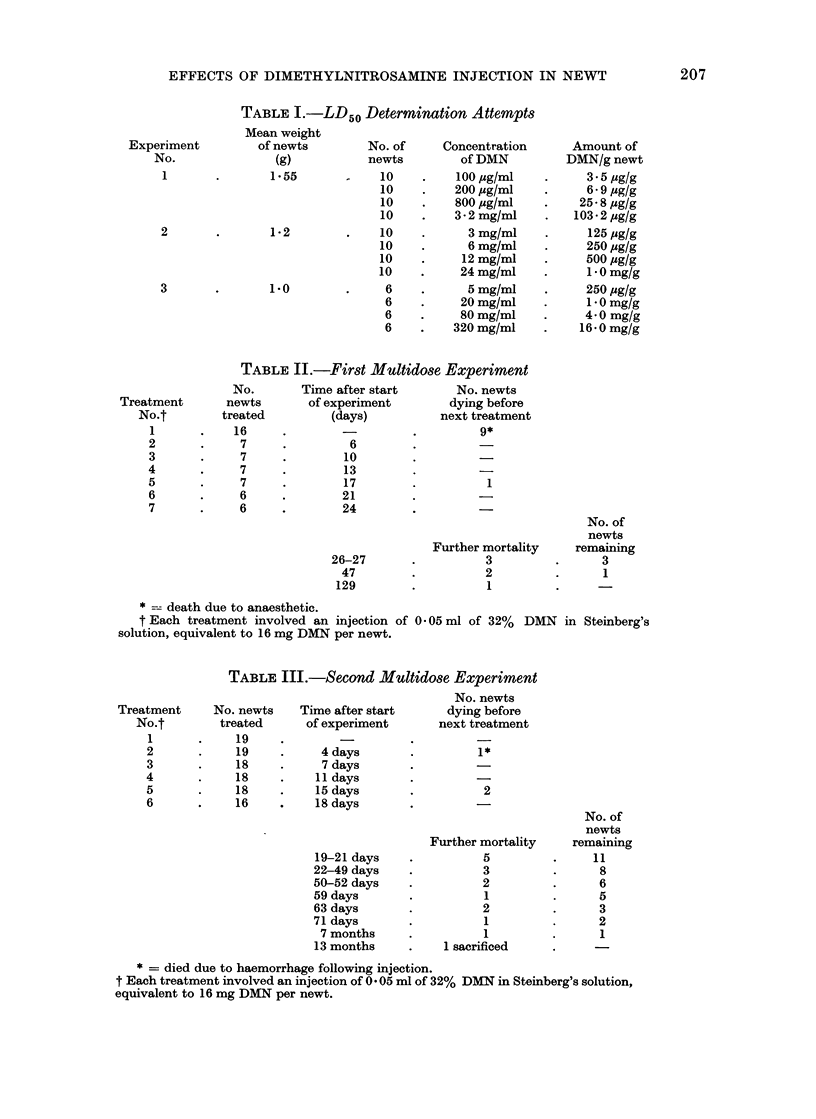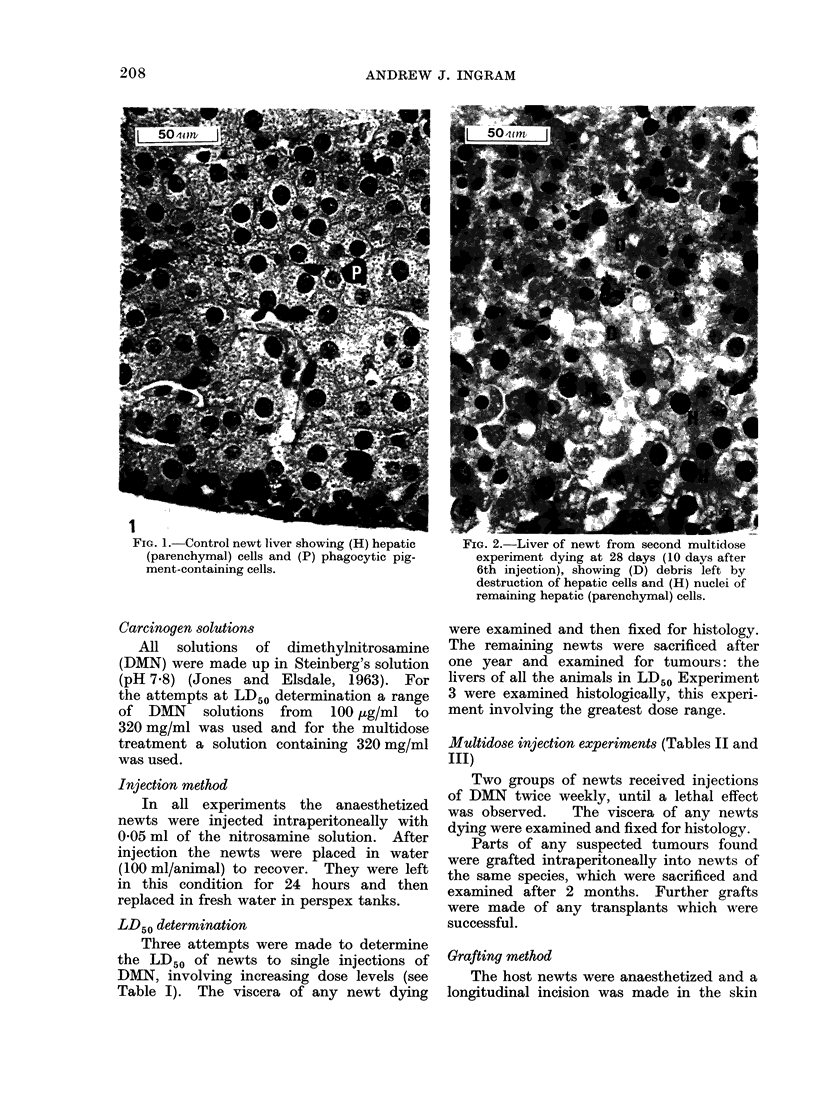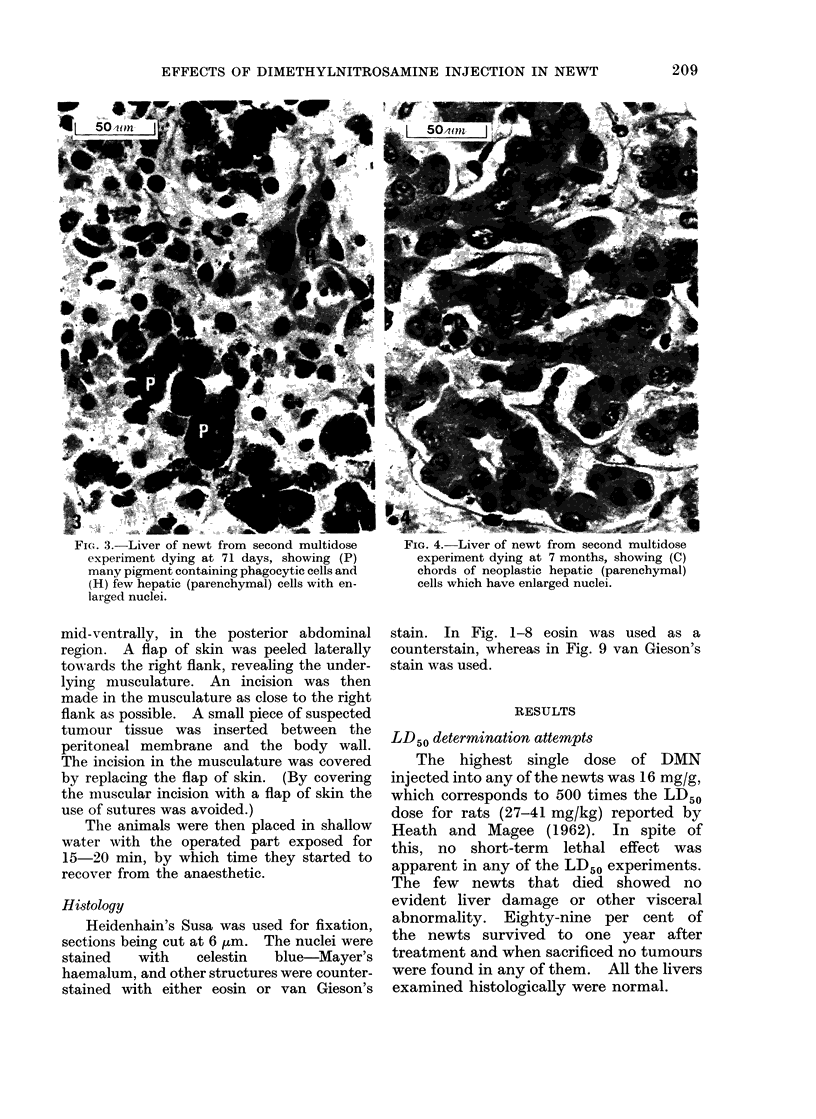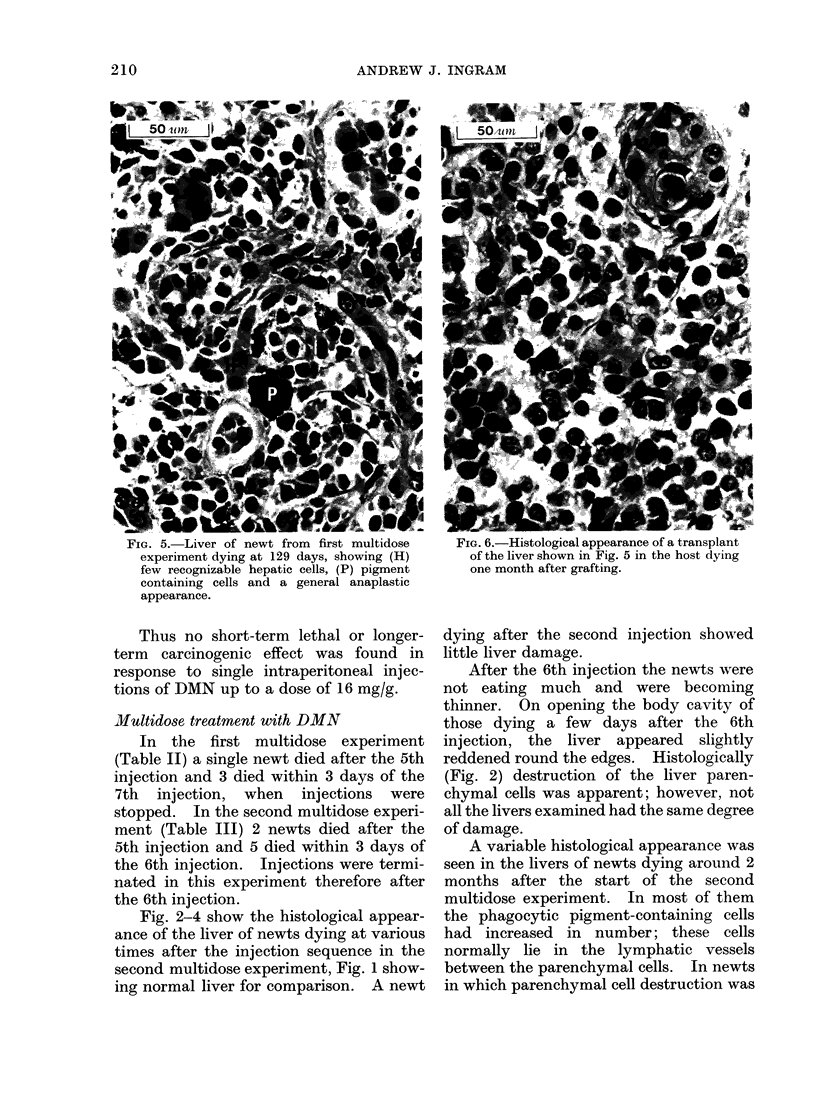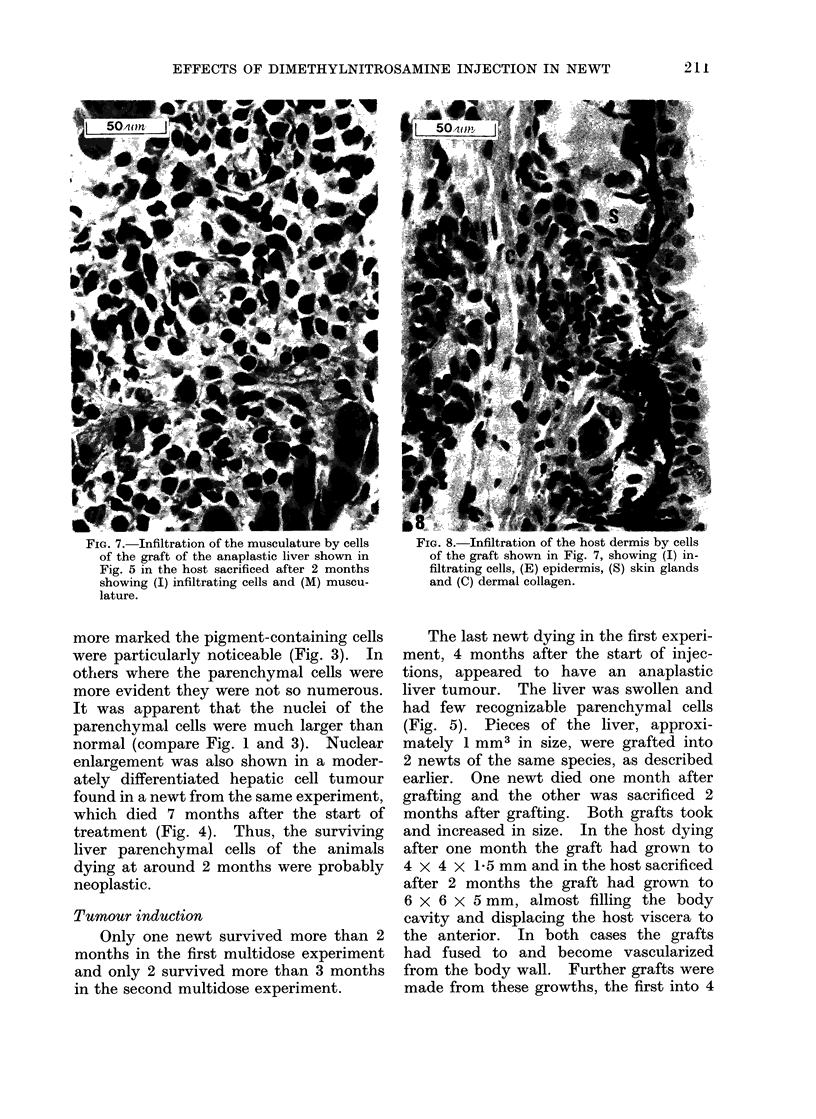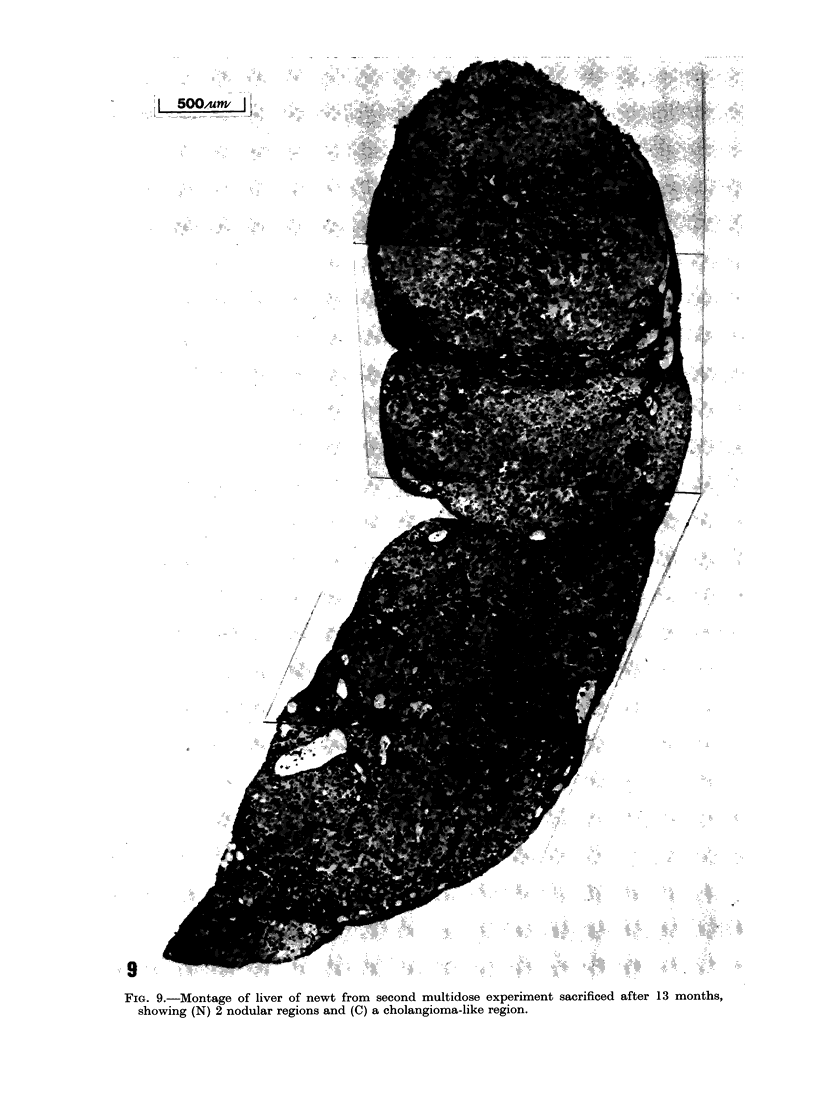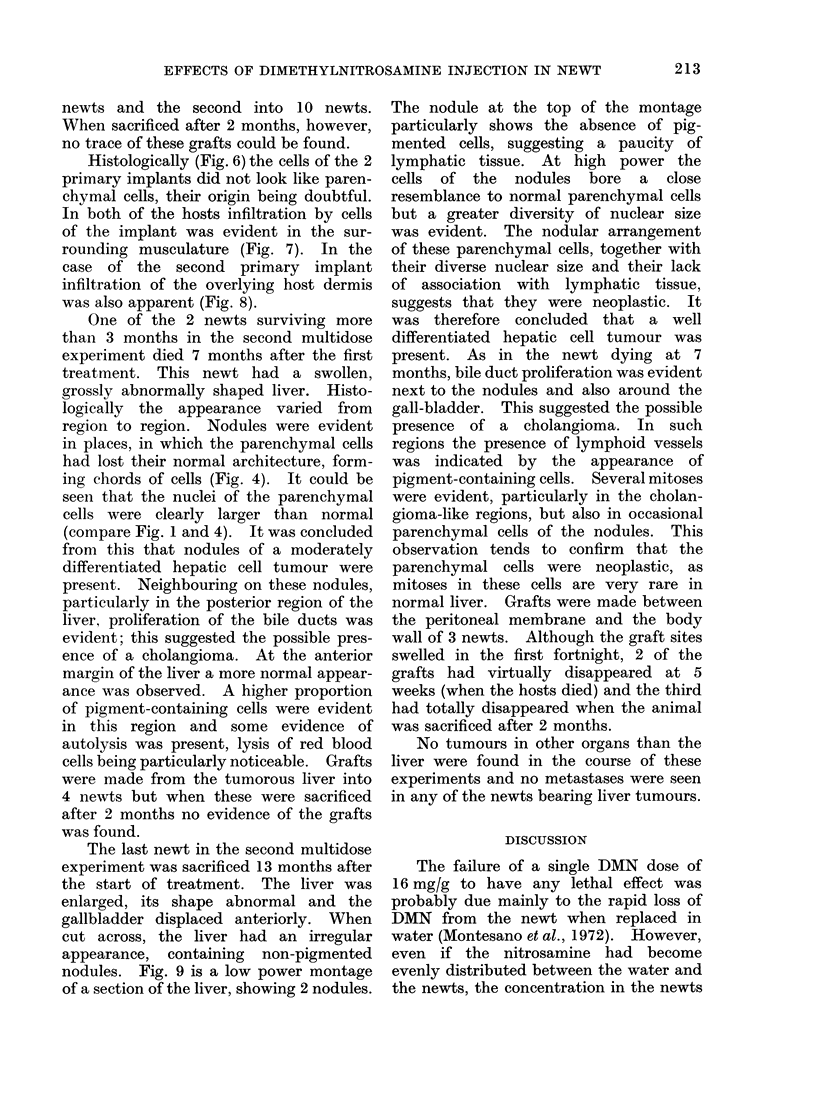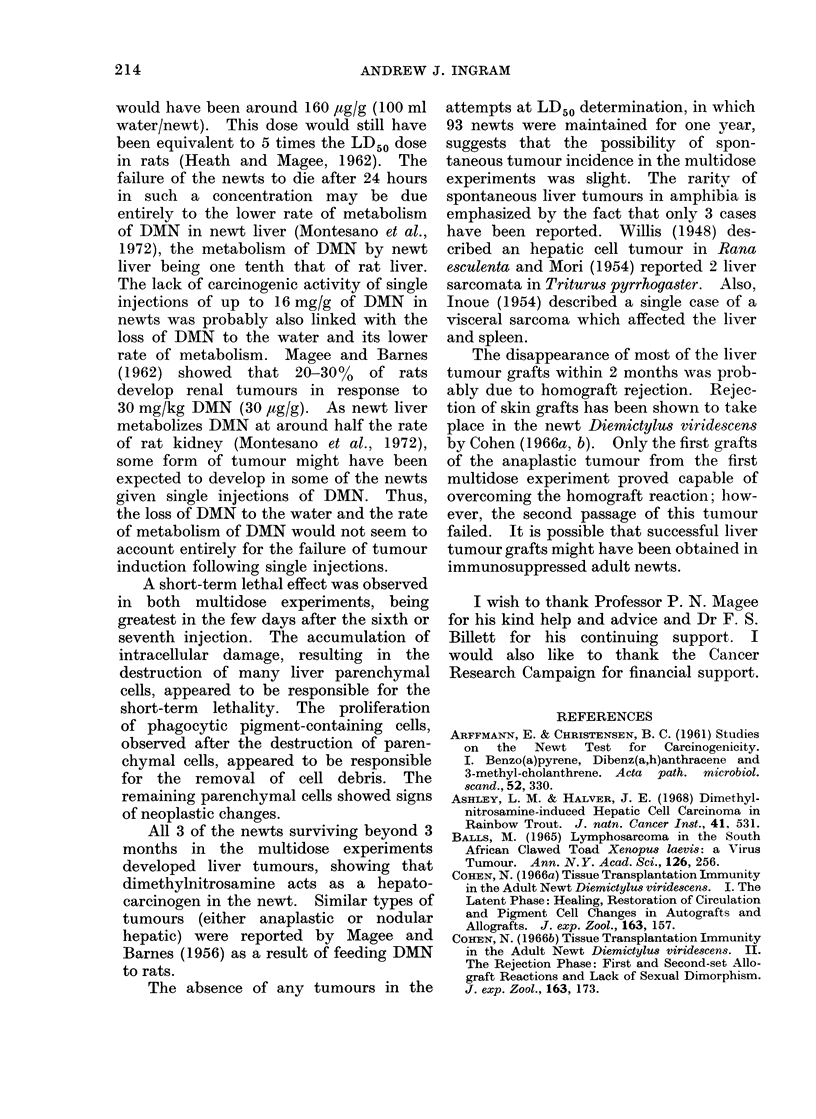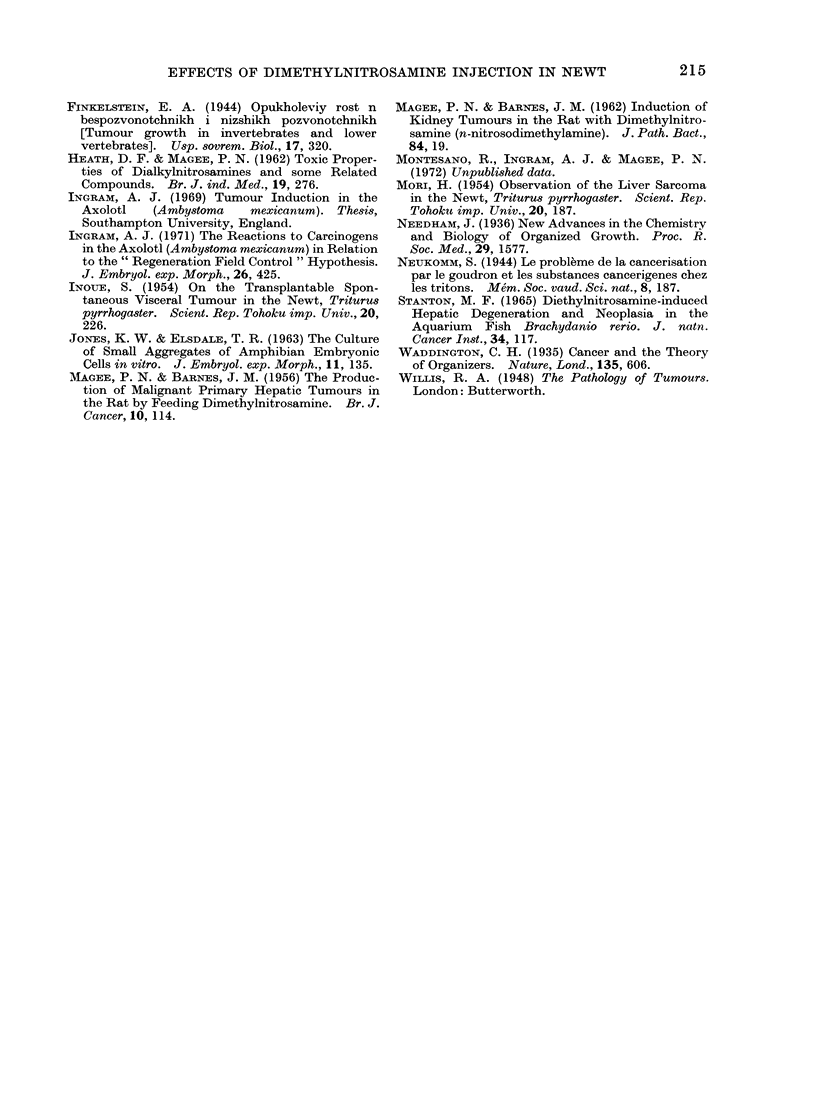# The Lethal and Hepatocarcinogenic Effects of Dimethylnitrosamine Injection in the Newt Triturus Helveticus

**DOI:** 10.1038/bjc.1972.28

**Published:** 1972-06

**Authors:** Andrew J. Ingram

## Abstract

**Images:**


*Br. J. Cancer* (1972) **26**, 206

### THE LETHAL AND HEPATOCARCINOGENIC EFFECTS OF DIMETHYLNITROSAMINE INJECTION IN THE NEWT *TRITURUS HELVETICUS*

ANDREW J. INGRAM

*Department of Zoology, University of Southampton, Southampton SO9 5NH*

Received for publication February 1972

**Summary.—Single intraperitoneal injections of dimethylnitrosamine (DMN) in concentrations of up to 16 mg/g body weight, failed to have any lethal effect in newts. This treatment also failed to induce tumours in newts maintained for one year after injection.**

**Six or 7 injections of 16 mg/g body weight of DMN, administered over a period of 3–4 weeks, gave rise to a short-term lethal effect due to liver necrosis and in the long term to liver tumours. The tumours induced were of similar type to nitrosamine-induced tumours in rats, one being an anaplastic liver tumour and the other nodular hepatic cell tumours.**

**Attempts to maintain the tumours by grafting failed, probably due to homograft rejection.**

MANY workers have emphasized the difficulty of inducing tumours in amphibia with polycyclic hydrocarbons (Finkelstein, 1944; Neukomm, 1944; Arffmann and Christensen, 1961; Ingram, 1969, 1971). Where authenticated tumours in amphibia have resulted from polycyclic hydrocarbon treatment, enhancement of latent oncogenic virus has been implicated in some cases (Balls, 1965). Explanations for the failure of tumour development in amphibia have been suggested (Waddington, 1935; Needham, 1936); however, the possibility that other carcinogens might be more suitable than polycyclic hydrocarbons has not been investigated.

Stanton (1965) and Ashley and Halver (1968) have shown that hepatomata can be induced in *Brachydanio rerio* (an aquarium fish), and the trout respectively by means of nitrosamines. Ashley and Halver administered the nitrosamine by adding it to the diet, whereas Stanton added it to the water.

As liver tumours can be induced in fish with nitrosamines, it seemed logical to test the carcinogenicity of these compounds in amphibia. The palmate newt *Triturus helveticus* was used for this investigation.

The aim of the first part of the work described here was to determine the $LD_{50}$ of the newt to single doses of dimethylnitrosamine injected intraperitoneally. The aim of the second part was to attempt to induce tumours by repeated injections of the same substance.

### MATERIALS AND METHODS

### *Animals*

Newts of the species *Triturus helveticus* were collected locally and kept in perspex tanks containing approximately 2 litres of water and a brick to enable them to leave the water. They were fed on chopped Tubifex worms every other day. The weight of the newts varied according to the time of the year and other factors, the mean weight of those used in these experiments being 1·25 g. The newts were anaesthetized with MS222 (tricaine methanesulphonate) before injection or grafting.

TABLE I.—*$LD_{50}$ Determination Attempts*

| Experiment No. | Mean weight of newts (g) | No. of newts | Concentration of DMN | Amount of DMN/g newt |
|---|---|---|---|---|
| 1 | 1·55 | 10 | 100 μg/ml | 3·5 μg/g |
| | | 10 | 200 μg/ml | 6·9 μg/g |
| | | 10 | 800 μg/ml | 25·8 μg/g |
| | | 10 | 3·2 mg/ml | 103·2 μg/g |
| 2 | 1·2 | 10 | 3 mg/ml | 125 μg/g |
| | | 10 | 6 mg/ml | 250 μg/g |
| | | 10 | 12 mg/ml | 500 μg/g |
| | | 10 | 24 mg/ml | 1·0 mg/g |
| 3 | 1·0 | 6 | 5 mg/ml | 250 μg/g |
| | | 6 | 20 mg/ml | 1·0 mg/g |
| | | 6 | 80 mg/ml | 4·0 mg/g |
| | | 6 | 320 mg/ml | 16·0 mg/g |

TABLE II.—*First Multidose Experiment*

| Treatment No.† | No. newts treated | Time after start of experiment (days) | No. newts dying before next treatment | |
|---|---|---|---|---|
| 1 | 16 | — | 9* | |
| 2 | 7 | 6 | — | |
| 3 | 7 | 10 | — | |
| 4 | 7 | 13 | — | |
| 5 | 7 | 17 | 1 | |
| 6 | 6 | 21 | — | |
| 7 | 6 | 24 | — | |
| | | | Further mortality | No. of newts remaining |
| | | 26–27 | 3 | 3 |
| | | 47 | 2 | 1 |
| | | 129 | 1 | — |

* = death due to anaesthetic.

† Each treatment involved an injection of 0·05 ml of 32% DMN in Steinberg's solution, equivalent to 16 mg DMN per newt.

TABLE III.—*Second Multidose Experiment*

| Treatment No.† | No. newts treated | Time after start of experiment | No. newts dying before next treatment | |
|---|---|---|---|---|
| 1 | 19 | — | — | |
| 2 | 19 | 4 days | 1* | |
| 3 | 18 | 7 days | — | |
| 4 | 18 | 11 days | — | |
| 5 | 18 | 15 days | 2 | |
| 6 | 16 | 18 days | — | |
| | | | Further mortality | No. of newts remaining |
| | | 19–21 days | 5 | 11 |
| | | 22–49 days | 3 | 8 |
| | | 50–52 days | 2 | 6 |
| | | 59 days | 1 | 5 |
| | | 63 days | 2 | 3 |
| | | 71 days | 1 | 2 |
| | | 7 months | 1 | 1 |
| | | 13 months | 1 sacrificed | — |

* = died due to haemorrhage following injection.

† Each treatment involved an injection of 0·05 ml of 32% DMN in Steinberg's solution, equivalent to 16 mg DMN per newt.

FIG. 1.—Control newt liver showing (H) hepatic (parenchymal) cells and (P) phagocytic pigment-containing cells.

FIG. 2.—Liver of newt from second multidose experiment dying at 28 days (10 days after 6th injection), showing (D) debris left by destruction of hepatic cells and (H) nuclei of remaining hepatic (parenchymal) cells.

*Carcinogen solutions*

All solutions of dimethylnitrosamine (DMN) were made up in Steinberg's solution (pH 7·8) (Jones and Elsdale, 1963). For the attempts at $LD_{50}$ determination a range of DMN solutions from 100 µg/ml to 320 mg/ml was used and for the multidose treatment a solution containing 320 mg/ml was used.

*Injection method*

In all experiments the anaesthetized newts were injected intraperitoneally with 0·05 ml of the nitrosamine solution. After injection the newts were placed in water (100 ml/animal) to recover. They were left in this condition for 24 hours and then replaced in fresh water in perspex tanks.

*$LD_{50}$ determination*

Three attempts were made to determine the $LD_{50}$ of newts to single injections of DMN, involving increasing dose levels (see Table I). The viscera of any newt dying were examined and then fixed for histology. The remaining newts were sacrificed after one year and examined for tumours: the livers of all the animals in $LD_{50}$ Experiment 3 were examined histologically, this experiment involving the greatest dose range.

*Multidose injection experiments* (Tables II and III)

Two groups of newts received injections of DMN twice weekly, until a lethal effect was observed. The viscera of any newts dying were examined and fixed for histology.

Parts of any suspected tumours found were grafted intraperitoneally into newts of the same species, which were sacrificed and examined after 2 months. Further grafts were made of any transplants which were successful.

*Grafting method*

The host newts were anaesthetized and a longitudinal incision was made in the skin

FIG. 3.—Liver of newt from second multidose experiment dying at 71 days, showing (P) many pigment containing phagocytic cells and (H) few hepatic (parenchymal) cells with enlarged nuclei.

FIG. 4.—Liver of newt from second multidose experiment dying at 7 months, showing (C) chords of neoplastic hepatic (parenchymal) cells which have enlarged nuclei.

mid-ventrally, in the posterior abdominal region. A flap of skin was peeled laterally towards the right flank, revealing the underlying musculature. An incision was then made in the musculature as close to the right flank as possible. A small piece of suspected tumour tissue was inserted between the peritoneal membrane and the body wall. The incision in the musculature was covered by replacing the flap of skin. (By covering the muscular incision with a flap of skin the use of sutures was avoided.)

The animals were then placed in shallow water with the operated part exposed for 15—20 min, by which time they started to recover from the anaesthetic.

*Histology*

Heidenhain's Susa was used for fixation, sections being cut at 6 $\mu$m. The nuclei were stained with celestin blue—Mayer's haemalum, and other structures were counterstained with either eosin or van Gieson's stain. In Fig. 1–8 eosin was used as a counterstain, whereas in Fig. 9 van Gieson's stain was used.

### RESULTS

*$LD_{50}$ determination attempts*

The highest single dose of DMN injected into any of the newts was 16 mg/g, which corresponds to 500 times the $LD_{50}$ dose for rats (27–41 mg/kg) reported by Heath and Magee (1962). In spite of this, no short-term lethal effect was apparent in any of the $LD_{50}$ experiments. The few newts that died showed no evident liver damage or other visceral abnormality. Eighty-nine per cent of the newts survived to one year after treatment and when sacrificed no tumours were found in any of them. All the livers examined histologically were normal.

FIG. 5.—Liver of newt from first multidose experiment dying at 129 days, showing (H) few recognizable hepatic cells, (P) pigment containing cells and a general anaplastic appearance.

FIG. 6.—Histological appearance of a transplant of the liver shown in Fig. 5 in the host dying one month after grafting.

Thus no short-term lethal or longer-term carcinogenic effect was found in response to single intraperitoneal injections of DMN up to a dose of 16 mg/g.

*Multidose treatment with DMN*

In the first multidose experiment (Table II) a single newt died after the 5th injection and 3 died within 3 days of the 7th injection, when injections were stopped. In the second multidose experiment (Table III) 2 newts died after the 5th injection and 5 died within 3 days of the 6th injection. Injections were terminated in this experiment therefore after the 6th injection.

Fig. 2–4 show the histological appearance of the liver of newts dying at various times after the injection sequence in the second multidose experiment, Fig. 1 showing normal liver for comparison. A newt dying after the second injection showed little liver damage.

After the 6th injection the newts were not eating much and were becoming thinner. On opening the body cavity of those dying a few days after the 6th injection, the liver appeared slightly reddened round the edges. Histologically (Fig. 2) destruction of the liver parenchymal cells was apparent; however, not all the livers examined had the same degree of damage.

A variable histological appearance was seen in the livers of newts dying around 2 months after the start of the second multidose experiment. In most of them the phagocytic pigment-containing cells had increased in number; these cells normally lie in the lymphatic vessels between the parenchymal cells. In newts in which parenchymal cell destruction was

FIG. 7.—Infiltration of the musculature by cells of the graft of the anaplastic liver shown in Fig. 5 in the host sacrificed after 2 months showing (I) infiltrating cells and (M) musculature.

FIG. 8.—Infiltration of the host dermis by cells of the graft shown in Fig. 7, showing (I) infiltrating cells, (E) epidermis, (S) skin glands and (C) dermal collagen.

more marked the pigment-containing cells were particularly noticeable (Fig. 3). In others where the parenchymal cells were more evident they were not so numerous. It was apparent that the nuclei of the parenchymal cells were much larger than normal (compare Fig. 1 and 3). Nuclear enlargement was also shown in a moderately differentiated hepatic cell tumour found in a newt from the same experiment, which died 7 months after the start of treatment (Fig. 4). Thus, the surviving liver parenchymal cells of the animals dying at around 2 months were probably neoplastic.

*Tumour induction*

Only one newt survived more than 2 months in the first multidose experiment and only 2 survived more than 3 months in the second multidose experiment.

The last newt dying in the first experiment, 4 months after the start of injections, appeared to have an anaplastic liver tumour. The liver was swollen and had few recognizable parenchymal cells (Fig. 5). Pieces of the liver, approximately 1 $mm^3$ in size, were grafted into 2 newts of the same species, as described earlier. One newt died one month after grafting and the other was sacrificed 2 months after grafting. Both grafts took and increased in size. In the host dying after one month the graft had grown to $4 \times 4 \times 1{\cdot}5$ mm and in the host sacrificed after 2 months the graft had grown to $6 \times 6 \times 5$ mm, almost filling the body cavity and displacing the host viscera to the anterior. In both cases the grafts had fused to and become vascularized from the body wall. Further grafts were made from these growths, the first into 4

FIG. 9.—Montage of liver of newt from second multidose experiment sacrificed after 13 months, showing (N) 2 nodular regions and (C) a cholangioma-like region.

newts and the second into 10 newts. When sacrificed after 2 months, however, no trace of these grafts could be found.

Histologically (Fig. 6) the cells of the 2 primary implants did not look like parenchymal cells, their origin being doubtful. In both of the hosts infiltration by cells of the implant was evident in the surrounding musculature (Fig. 7). In the case of the second primary implant infiltration of the overlying host dermis was also apparent (Fig. 8).

One of the 2 newts surviving more than 3 months in the second multidose experiment died 7 months after the first treatment. This newt had a swollen, grossly abnormally shaped liver. Histologically the appearance varied from region to region. Nodules were evident in places, in which the parenchymal cells had lost their normal architecture, forming chords of cells (Fig. 4). It could be seen that the nuclei of the parenchymal cells were clearly larger than normal (compare Fig. 1 and 4). It was concluded from this that nodules of a moderately differentiated hepatic cell tumour were present. Neighbouring on these nodules, particularly in the posterior region of the liver, proliferation of the bile ducts was evident; this suggested the possible presence of a cholangioma. At the anterior margin of the liver a more normal appearance was observed. A higher proportion of pigment-containing cells were evident in this region and some evidence of autolysis was present, lysis of red blood cells being particularly noticeable. Grafts were made from the tumorous liver into 4 newts but when these were sacrificed after 2 months no evidence of the grafts was found.

The last newt in the second multidose experiment was sacrificed 13 months after the start of treatment. The liver was enlarged, its shape abnormal and the gallbladder displaced anteriorly. When cut across, the liver had an irregular appearance, containing non-pigmented nodules. Fig. 9 is a low power montage of a section of the liver, showing 2 nodules. The nodule at the top of the montage particularly shows the absence of pigmented cells, suggesting a paucity of lymphatic tissue. At high power the cells of the nodules bore a close resemblance to normal parenchymal cells but a greater diversity of nuclear size was evident. The nodular arrangement of these parenchymal cells, together with their diverse nuclear size and their lack of association with lymphatic tissue, suggests that they were neoplastic. It was therefore concluded that a well differentiated hepatic cell tumour was present. As in the newt dying at 7 months, bile duct proliferation was evident next to the nodules and also around the gall-bladder. This suggested the possible presence of a cholangioma. In such regions the presence of lymphoid vessels was indicated by the appearance of pigment-containing cells. Several mitoses were evident, particularly in the cholangioma-like regions, but also in occasional parenchymal cells of the nodules. This observation tends to confirm that the parenchymal cells were neoplastic, as mitoses in these cells are very rare in normal liver. Grafts were made between the peritoneal membrane and the body wall of 3 newts. Although the graft sites swelled in the first fortnight, 2 of the grafts had virtually disappeared at 5 weeks (when the hosts died) and the third had totally disappeared when the animal was sacrificed after 2 months.

No tumours in other organs than the liver were found in the course of these experiments and no metastases were seen in any of the newts bearing liver tumours.

### DISCUSSION

The failure of a single DMN dose of 16 mg/g to have any lethal effect was probably due mainly to the rapid loss of DMN from the newt when replaced in water (Montesano *et al.*, 1972). However, even if the nitrosamine had become evenly distributed between the water and the newts, the concentration in the newts would have been around 160 $\mu$g/g (100 ml water/newt). This dose would still have been equivalent to 5 times the $LD_{50}$ dose in rats (Heath and Magee, 1962). The failure of the newts to die after 24 hours in such a concentration may be due entirely to the lower rate of metabolism of DMN in newt liver (Montesano *et al.*, 1972), the metabolism of DMN by newt liver being one tenth that of rat liver. The lack of carcinogenic activity of single injections of up to 16 mg/g of DMN in newts was probably also linked with the loss of DMN to the water and its lower rate of metabolism. Magee and Barnes (1962) showed that 20–30% of rats develop renal tumours in response to 30 mg/kg DMN (30 $\mu$g/g). As newt liver metabolizes DMN at around half the rate of rat kidney (Montesano *et al.*, 1972), some form of tumour might have been expected to develop in some of the newts given single injections of DMN. Thus, the loss of DMN to the water and the rate of metabolism of DMN would not seem to account entirely for the failure of tumour induction following single injections.

A short-term lethal effect was observed in both multidose experiments, being greatest in the few days after the sixth or seventh injection. The accumulation of intracellular damage, resulting in the destruction of many liver parenchymal cells, appeared to be responsible for the short-term lethality. The proliferation of phagocytic pigment-containing cells, observed after the destruction of parenchymal cells, appeared to be responsible for the removal of cell debris. The remaining parenchymal cells showed signs of neoplastic changes.

All 3 of the newts surviving beyond 3 months in the multidose experiments developed liver tumours, showing that dimethylnitrosamine acts as a hepatocarcinogen in the newt. Similar types of tumours (either anaplastic or nodular hepatic) were reported by Magee and Barnes (1956) as a result of feeding DMN to rats.

The absence of any tumours in the attempts at $LD_{50}$ determination, in which 93 newts were maintained for one year, suggests that the possibility of spontaneous tumour incidence in the multidose experiments was slight. The rarity of spontaneous liver tumours in amphibia is emphasized by the fact that only 3 cases have been reported. Willis (1948) described an hepatic cell tumour in *Rana esculenta* and Mori (1954) reported 2 liver sarcomata in *Triturus pyrrhogaster*. Also, Inoue (1954) described a single case of a visceral sarcoma which affected the liver and spleen.

The disappearance of most of the liver tumour grafts within 2 months was probably due to homograft rejection. Rejection of skin grafts has been shown to take place in the newt *Diemictylus viridescens* by Cohen (1966*a*, *b*). Only the first grafts of the anaplastic tumour from the first multidose experiment proved capable of overcoming the homograft reaction; however, the second passage of this tumour failed. It is possible that successful liver tumour grafts might have been obtained in immunosuppressed adult newts.

I wish to thank Professor P. N. Magee for his kind help and advice and Dr F. S. Billett for his continuing support. I would also like to thank the Cancer Research Campaign for financial support.

### REFERENCES

Arffmann, E. & Christensen, B. C. (1961) Studies on the Newt Test for Carcinogenicity. I. Benzo(a)pyrene, Dibenz(a,h)anthracene and 3-methyl-cholanthrene. *Acta path. microbiol. scand.*, **52**, 330.

Ashley, L. M. & Halver, J. E. (1968) Dimethylnitrosamine-induced Hepatic Cell Carcinoma in Rainbow Trout. *J. natn. Cancer Inst.*, **41**, 531.

Balls, M. (1965) Lymphosarcoma in the South African Clawed Toad *Xenopus laevis*: a Virus Tumour. *Ann. N.Y. Acad. Sci.*, **126**, 256.

Cohen, N. (1966*a*) Tissue Transplantation Immunity in the Adult Newt *Diemictylus viridescens*. I. The Latent Phase: Healing, Restoration of Circulation and Pigment Cell Changes in Autografts and Allografts. *J. exp. Zool.*, **163**, 157.

Cohen, N. (1966*b*) Tissue Transplantation Immunity in the Adult Newt *Diemictylus viridescens*. II. The Rejection Phase: First and Second-set Allograft Reactions and Lack of Sexual Dimorphism. *J. exp. Zool.*, **163**, 173.

FINKELSTEIN, E. A. (1944) Opukholeviy rost n bespozvonotchnikh i nizshikh pozvonotchnikh [Tumour growth in invertebrates and lower vertebrates]. *Usp. sovrem. Biol.*, **17**, 320.

HEATH, D. F. & MAGEE, P. N. (1962) Toxic Properties of Dialkylnitrosamines and some Related Compounds. *Br. J. ind. Med.*, **19**, 276.

INGRAM, A. J. (1969) Tumour Induction in the Axolotl (*Ambystoma mexicanum*). *Thesis*, Southampton University, England.

INGRAM, A. J. (1971) The Reactions to Carcinogens in the Axolotl (*Ambystoma mexicanum*) in Relation to the " Regeneration Field Control " Hypothesis. *J. Embryol. exp. Morph.*, **26**, 425.

INOUE, S. (1954) On the Transplantable Spontaneous Visceral Tumour in the Newt, *Triturus pyrrhogaster*. *Scient. Rep. Tohoku imp. Univ.*, **20**, 226.

JONES, K. W. & ELSDALE, T. R. (1963) The Culture of Small Aggregates of Amphibian Embryonic Cells *in vitro*. *J. Embryol. exp. Morph.*, **11**, 135.

MAGEE, P. N. & BARNES, J. M. (1956) The Production of Malignant Primary Hepatic Tumours in the Rat by Feeding Dimethylnitrosamine. *Br. J. Cancer*, **10**, 114.

MAGEE, P. N. & BARNES, J. M. (1962) Induction of Kidney Tumours in the Rat with Dimethylnitrosamine (*n*-nitrosodimethylamine). *J. Path. Bact.*, **84**, 19.

MONTESANO, R., INGRAM, A. J. & MAGEE, P. N. (1972) *Unpublished data.*

MORI, H. (1954) Observation of the Liver Sarcoma in the Newt, *Triturus pyrrhogaster*. *Scient. Rep. Tohoku imp. Univ.*, **20**, 187.

NEEDHAM, J. (1936) New Advances in the Chemistry and Biology of Organized Growth. *Proc. R. Soc. Med.*, **29**, 1577.

NEUKOMM, S. (1944) Le problème de la cancerisation par le goudron et les substances cancerigenes chez les tritons. *Mém. Soc. vaud. Sci. nat.*, **8**, 187.

STANTON, M. F. (1965) Diethylnitrosamine-induced Hepatic Degeneration and Neoplasia in the Aquarium Fish *Brachydanio rerio*. *J. natn. Cancer Inst.*, **34**, 117.

WADDINGTON, C. H. (1935) Cancer and the Theory of Organizers. *Nature, Lond.*, **135**, 606.

WILLIS, R. A. (1948) *The Pathology of Tumours.* London: Butterworth.